# Australian Bogong moths *Agrotis infusa* (Lepidoptera: Noctuidae), 1951–2020: decline and crash

**DOI:** 10.1111/aen.12517

**Published:** 2020-12-18

**Authors:** Ken Green, Peter Caley, Monika Baker, David Dreyer, Jesse Wallace, Eric Warrant

**Affiliations:** ^1^ Australian National University College of Asia and the Pacific Canberra ACT 2601 Australia; ^2^ Data 61, Commonwealth Scientific and Industrial Research Organisation Canberra ACT 2601 Australia; ^3^ Bayside Melbourne Vic 3193 Australia; ^4^ Lund Vision Group, Department of Biology University of Lund Sölvegatan 35 Lund S‐22362 Sweden; ^5^ Research School of Biology Australian National University Canberra ACT 2601 Australia; ^6^ Division of Information, Technology and Development University of South Australia Adelaide SA 5001 Australia

**Keywords:** *Agrotis infusa*, alpine ecosystem, Bogong moth, climate change, historical record, insect decline, migration

## Abstract

The Bogong moth *Agrotis infusa* is well known for its remarkable long‐distance migration – a return journey from the plains of southeast Australia to the Australian Alps – as well as for its cultural significance for Indigenous Australians. Each spring, as many as four billion moths are estimated to arrive in the Australian Alps to aestivate in cool mountain caves and in boulder fields, bringing with them a massive annual influx of energy and nutrients critical for the health of the alpine ecosystem. However, a massive decline in moths present at their aestivation sites has occurred over the past 3 years, with only a few individuals present where hundreds of thousands could earlier be found. In order to understand the possible sources of decline, we analysed historical records of Bogong moth numbers at aestivation sites in the Australian Alps, including observations on Mt. Gingera (NSW) in the early 1950s, observations from 1980 onwards in the Snowy Mountains (NSW) and an almost‐unbroken series of observations each summer over the past 53 years in three caves at different elevations on Mt. Buffalo (Victoria). This analysis shows that moth numbers were probably steady from 1951 until about 1980, fluctuated and slowly fell from then until 2016 and dramatically crashed in 2017. In the Murray–Darling Basin, the main winter breeding ground of Bogong moths, changes in farming practices, such as increasing land clearing for crops (which has removed around a quarter of a billion moths annually from the mountains compared to pre‐European levels), has probably driven some of the decline in Bogong moth numbers observed from 1980 to 2016. The impact of insecticide remains unclear and is in urgent need of further study. Even though we found little evidence that increasing global temperatures per se are responsible for the Bogong moth decline, the Australian climate has nonetheless become drier and warmer over past decades, possibly hampering the survival of immature stages in the breeding areas and confining adult aestivation to gradually higher elevations. The crash in moth numbers from 2017 is most likely due to the recent severe drought in the moth's breeding grounds.

## Introduction

The Bogong moth *Agrotis infusa* is well known for its remarkable long‐distance migration (Common 1952, 1954; Warrant *et al.* 2016; Dreyer *et al.* 2018) and its cultural significance for Indigenous Australians (Flood 1980, 1996). Newly emerged Bogong moths escape the increasing heat of the breeding grounds in western and north‐western New South Wales (NSW), southern Queensland, western Victoria and eastern South Australia, by migrating during spring to the mountain regions of south‐eastern Australia, a journey of up to 1000 km. Once at high elevation in the mountains, moths eventually seek out the shelter of cool caves, and the deep hollows and crevices of boulder fields, where they aestivate in vast numbers until autumn (aestivation is a state of dormancy similar to hibernation but instead occurring in summer). Bogong moths – which tile the cave walls at a density of around 17 000 individuals per square metre – are capable of aestivating for up to 4 months (Common 1954). Following this period, the same individuals that arrived months earlier return to the breeding grounds where they mate lay their eggs and die (Common 1952, 1954; Warrant *et al.* 2016). The larvae then develop during the coming winter, being found in soils across a variety of land types, where they feed on the stems of broad‐leafed plants at ground level. Pupation occurs in late winter, about 2–3 cm underground, with eclosion occurring during the following spring (Common 1981; Warrant *et al.* 2016).

Bogong moth aggregations in the mountains were first reported in NSW by Bennett (1834) and later by Scott (1873), but no satisfactory explanation for the moth assemblages was offered until that by Common (1952). In this and his later paper (Common 1954), Common argues that the reason for migration, and subsequent aestivation, is the increasingly unfavourable conditions of the breeding grounds. Before Bogong moths begin to congregate at high elevations in the mountains to aestivate over the summer, a number of lower sites are usually occupied temporarily (Common 1954), most often at lower subalpine elevations in the mountains. However, in some years, these lower sites can also occur at a great distance from the mountain aestivation sites, for instance in houses, Parliament House in Canberra or other similar locations (such as sports grounds) where large bright lights attract them. These are ‘accidental’ sites, however, and away from lights Bogong moths may use a variety of ground‐based sites such as dead trees. Moths have even been observed to congregate in small animal traps that have been used in dietary studies of mammals. The dates of the first Bogong moth arrivals at these lower sites can vary depending on the year and the location.

Bogong moths eventually cease feeding and ascend to the caves to aestivate. The moth's aestivation sites include a variety of granitic locations in the Brindabella Ranges of the Australian Capital Territory (ACT) and NSW, and in the Bogong Peaks and Snowy Mountains of NSW, and at a number of granitic sites in Victoria, including Mt. Buffalo and the Jaithmathangs. These mountains are characterised by granitic outcrops and boulder fields (Common 1954) and include the highest sites where Bogong moths can aestivate and be observed throughout the summer. The basalt boulder fields of Victoria (e.g. on the Bogong and Hotham High Plains) also provide aestivation sites (Mansergh 1988; Mansergh and Heinze 2019).

The first 30 years of Bogong moth research (1950–1980) established the numbers of Bogong moths at a time when climate change was becoming an issue of concern, with the global mean surface temperature having now been established relative to the 1951–1980 mean (ASA Goddard Institute for Space Studies). In 60 years of recorded measurements from the central Snowy Mountains, there has been a steady decrease in the maximum depth of the snowpack, and a shortening of the mean duration of the snowpack, by 18.5 days. These changes have resulted from a mean temperature increase of 0.36°C per decade (Sánchez‐Bayo and Green 2013), which may have also impacted Bogong moth numbers.

Although there has been some suspicion that moth numbers at aestivation sites have declined over the years (Flood 1980; but see a discussion in Blakers 1980), records of Bogong moth abundance collated and/or collected by the authors over nearly 70 years (detailed further) indicate that we have now experienced a sudden and major decline (Mansergh and Heinze 2019). This occurred in the summer of 2017–2018, the worst summer ever recorded for Bogong moth numbers, with very few moths seen in a continuously monitored cave on an unnamed peak above Dead Horse Gap near Thredbo (2020 m), a boulder outcrop near South Ramshead in the Kosciuszko Main Range of NSW (which we, the first author's co‐authors, in recognition of his long history of visits to the site, have dubbed ‘Ken Green Bogong’). In January 2019, no moths at all were found in this cave. This situation was mirrored in caves at a number of other mountain locations that had been studied over many years: Mt. Gingera (NSW/ACT), Mt. Morgan (NSW), Mt. Buffalo (Vic.) and the Bogong and Hotham High Plains (Vic.) (Mansergh and Heinze 2019). Increases in moth numbers were observed in the NSW and ACT caves during the summer of 2019–2020, but the population was still depleted. This rapid decline probably had a tremendous impact on the health of the alpine ecosystem of south‐eastern Australia as the massive influx of energy and nutrients that Bogong moths provide via their summer migration to the mountains (Green 2011) had evaporated. The aim of this review is to examine the timing and causes of the dramatic decline in Bogong moth numbers in the Australian Alps.

To examine this decline, we re‐examined data collected in the early 1950s by Ian Common from Mt. Gingera, on the border between NSW and the ACT (Common 1952, 1954), and analysed our own data collected from a number of locations over the past 53 years. These data include notes on Bogong moth arrivals and moth activity over four decades in 13 regularly surveyed aestivation sites across the Snowy Mountains of NSW (KG: personal field notebooks and PhD data), data from over four decades of studies of the diet of foxes in the Snowy Mountains (KG: biannual 6 km transects), Bogong moth activity on Mt. Gingera (PC: 37 separate aestivation sites, observed approximately bi‐weekly from September to May since 2014, excluding the summers of 2016–2017 and 2017–2018), records of Bogong moth numbers from aestivation sites at three elevations on Mt. Buffalo in Victoria over the past 53 years (MB), and moth collections from the Snowy Mountains, the Brindabella Ranges and the Mt. Kaputar National Park (northern NSW) for studies of Bogong moth migration (EW, DD and JW: each year during spring and autumn since 2010).

## Observations of Bogong Moth Numbers in Southeast Australia

### Larvae in the breeding grounds

**Fig. 1:**
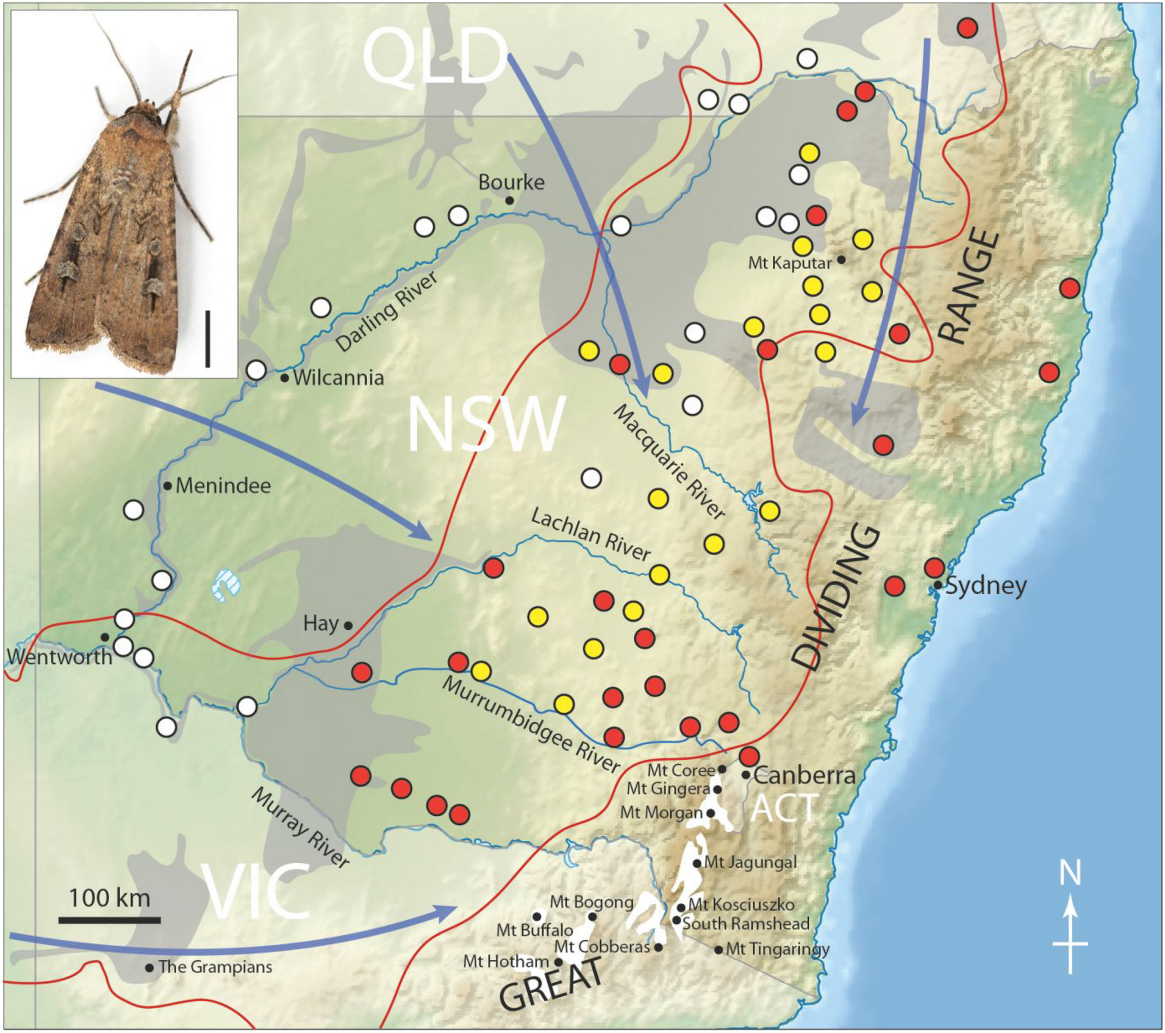
Map of south‐eastern Australia showing the locations where larvae have been collected by Froggatt (1900, *yellow circles*), Common (1954, *red circles*) and Green (2008, *white circles*). Also shown are the names of the major rivers in NSW, and the locations of various towns and mountain peaks mentioned in the text. The area of broadacre cropping farms across south eastern Australia during 2014–2015 is delineated by the *red line* (from Hughes, 2017), while areas of grey cracking clays are shown in *grey*. Bogong moths fly towards the Australian Alps (*white areas*, elevations > 1500 m) from various regions of southeast Australia, from as far distant as eastern South Australia, western Victoria, western and northwest New South Wales and southern and southeast Queensland (*blue arrows*). Adapted from Warrant *et al.* (2016), after Green (2008). Inset: The Australian Bogong moth *Agrotis infusa* (Boisduval, 1832). Scale bar = 5 mm. Reproduced with the kind permission of the photographer; Ajay Narendra, Macquarie University, Australia.

Even though Bogong moths can be recorded elsewhere, regions of inland south‐eastern Australia that possess grey cracking clays are the most common breeding grounds (Common 1954). We measured these areas in NSW and Queensland to obtain an approximate area of 101 180 km^2^. Bogong moth larvae were surveyed from 29 sites in 2001, primarily in locations with grey cracking clays (Green 2008 – Fig. 1). This resulted in a calculated density of 0.78 Bogong moth larvae per 10 m^2^, (or 78 000 larvae km^−2^) giving a total of around 7.9 billion larvae within the region of grey cracking clays of NSW and Queensland. Adult Bogong moth numbers were also calculated for the Snowy Mountains, based on calculations of predation and other sources of mortality (Green 2010b) together with a percentage survival from Blakers (1980), to give a total number of 2.2 billion adult Bogong moths. According to the Interim Biogeographic Regionalisation for Australia (IBRA), developed by the Australian Government, the Australian Alps consists of two subregions: the Snowy Mountains (IBRA code AUA01: area 713 114 Ha) and the Victorian Alps (IBRA code AUA02: area 519,866 Ha). The areas of these two subregions indicate that the Victorian Alps cover around 73% of the area of the Snowy Mountains (the white alpine regions shown in Fig. 1). If we assume that moths are evenly spread over both alpine regions, this would imply that around 1.6 billion adult moths are present in Victoria, giving a total of 3.8 billion Bogong moths overall in the Australian Alps. If the figures above (7.9 and 3.8 billion) are reliable, this would suggest an attrition of ~50% from Bogong moth larvae to post‐migration adults. There are occasional records of Bogong moth adults occurring far from the Australian Alps during the summer (and at other times of the year), but these moths are in low numbers, and none of them have been recorded aestivating in any other cave system outside the Australian Alps, either in NSW or Victoria. These moths may represent individuals from small non‐migratory populations (Common 1954; Warrant *et al.* 2016).

### Adults during migration

During migration, Bogong moths are apparently not attracted to caves along the route, and in areas where moth migration is commonly recorded, for example, on Mt. Kaputar (EW), no evidence of Bogong moth aestivation was found in the bat‐free caves around the summit (at 1510 m). However, some of these caves housed other species of moths, such as the Granny's Cloak moth *Speiredonia spectans* (investigations in caves near the summit by KG in October 2015: Warrant *et al.* 2016).

As part of studies of the Bogong moth migration, adult moths were collected at light traps during spring on Mt. Kaputar in northern NSW. These moths were flying south‐southwest (Dreyer *et al.* 2018) so had most likely originated in south‐eastern Queensland (Fig. 1). Moths were captured every third night with hundreds being caught during most trapping sessions from 2011 to 2015. In 2017, trapping recommenced at Mt. Kaputar and in the first 10 days of November, only 70 moths were caught. In 2018, around 100 moths were caught over a 2‐week period in October. Trapping of adult moths – when moths were presumably at the beginning of their migration – was also undertaken at various locations along the Darling River in western NSW in mid‐October 2016, with 41 moths taken over two nights with a light trap near Wentworth and 42 moths on a single night at Wilcannia. Some nights had adverse weather conditions (very low temperatures and/or rain), and on these nights, no moths were trapped. The winter and spring of 2016 were very wet across NSW with major flooding occurring in several regions of the central west – the grasslands of western NSW were dense and lush during that particular spring.

In the following year – 2017, the first year of the recent current extreme drought – light trapping of adults was again undertaken on the Western Plains during the last 2 weeks of October – at Hay, Wentworth, Wilcannia and Bourke. Of the 44 Bogong moths caught over that 2‐week period, 42 of them were caught over several nights at Hay. Trapping was again undertaken for the entire month of October in 2018 near Hay, Wentworth, and west of the Grampians in Victoria, with even lower numbers than the previous year – around 30 moths in almost as many nights.

Thus, although light‐trapping observations of adult migrants, many hundreds of kilometres from the Australian Alps, have only been made since 2010, Bogong moths were highly abundant from 2010 until 2016 but suffered a dramatic drop in their numbers from 2017 to 2020. This implies that the crash in Bogong moth numbers observed in the Australian Alps over the same period was primarily due to losses in their breeding grounds and not to conditions prevailing at their high elevation aestivation sites (albeit some further losses may also have occurred in the mountains).

### Arrival of adults in the mountains

As Bogong moths arrive in the vicinity of the caves in the Australian Alps, they first arrive at lower elevations where they shelter and feed until conditions improve sufficiently at higher elevations to ascend to their aestivation caves (Common 1954; Warrant *et al.* 2016). In the Brindabella Ranges, ‘temporary camps’ are occupied at lower elevations between 1220 and 1580 m (Common 1954). They have also been observed in spring at lower elevations in northern parts of the Snowy Mountains, notably at Happy Jacks (1240 m) and Snow Ridge (1495 m) (Gibson *et al.* 2018).

During weekly surveys of several aestivation sites on Mt. Gingera, during two summers (2014–2015 and 2015–2016), Caley and Welvaert (2018) found that moths arrived approximately 1 month earlier (in the first week of October) and left 1 month earlier (during early March) than they did during the surveys conducted by Ian Common during the early 1950s (arrival in mid‐November, and departure during early April: Common 1954). This is consistent with the recent decades of warmer climate, but further surveys are needed to confirm this trend.

When the Bogong moths arrive at these lower elevations, it is likely that snow still blocks the entrances to their aestivation caves at higher elevations. However, Bogong moths can shelter at higher sites without snow blockage, even where sub‐zero temperatures have been recorded using temperature loggers (e.g. in a subalpine tree stump at 1860 m on 14 October 2001 and in an alpine cave at 1940 m on 10 November 2001). Most moths, however, are likely to be elsewhere, feeding or waiting at lower elevations to move up hill to the aestivation sites. The lack of suitable sites at these elevations makes it difficult to obtain data on these locations. However, predators such as mountain pygmy possums and foxes provide a suitable source of data. The diet of foxes was studied along a transect from 1500–1800 m elevation at Schlink Pass in the Kosciuszko National Park (Green and Osborne 1981), with fox scats collected monthly from December 1978 to February 1980 (although these were not studied according to elevation). Of the 1159 scats collected and analysed, 151 contained only Bogong moth remains (Green and Osborne 1981). The number of Bogong moths present in scats increased from October to peak in December to January, both in 1978 and 1979, and declined to zero in May 1979 (Green and Osborne 1981). This indicates that moth numbers rose early post snow‐thaw below the elevation of Schlink Pass (1800 m). As moth numbers fell in scats from the 1500–1800 m of elevation band after their December peak, foxes were probably still feeding on moths at elevations above 1800 m – at aestivating sites such as that at Dicky Cooper Bogong (1985 m, above Schlink Pass) – possibly defecating up there rather than at lower elevations where scats were being studied.

**Fig. 2:**
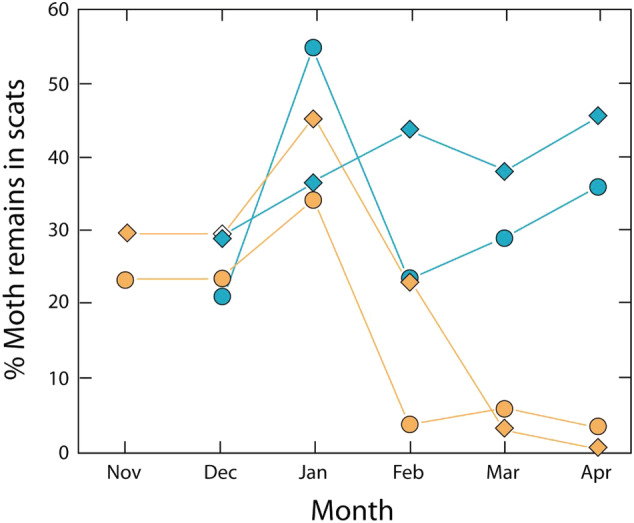
Mean proportion of individual fox scats consisting of Bogong moth remains at subalpine (*orange lines and symbols*) and alpine altitudes (*blue lines and symbols*) over two summer seasons: 1996–1997 (*diamonds*) and 1997–1998 (*circles*). Redrawn from Green (2010b).

Fox diet was also studied at well separated alpine and subalpine study sites (Green 2010b). This showed that Bogong moth numbers in scats at subalpine sites again peaked in December to January, declining rapidly from January with low numbers thereafter (Green 2010b). By contrast, at alpine sites, numbers remained high and in fact increased (Green 2010b) (Fig. 2). The year 1996–1997 showed a later arrival than in 1997–1998 but received more Bogong moth arrivals later in the season (Fig. 2). This indicates that Bogong moths were moving to higher elevations as summer progressed, and the same pattern can be observed from studies of the scats of the Mountain pygmy‐possum *B* *urramys* *parvus* (Gibson *et al.* 2018, Table 1).

**Table 1:** Maximal shade temperatures *T* in boulder fields on the Main Range of the Snowy Mountains at four elevations during the snow‐free season^†^, together with proportion of Bogong moths in Mountain pygmy possum (*Burramys parvus*) scats recorded over 17 years in different seasons^‡^ and frequency of occurrence of Bogong moths in scats of Dusky antechinus (*Antechinus swainsonii*) in 1981–1987^§^ ^,^ ^¶^

Location	Mean T (°C)	Late Spring	Late Summer
Charlotte Pass, 1755 m	12.3 ± 2.9	30%^‡^	≈1%^‡^
South Ramshead, 2000 m	10.7 ± 3.5	62%^§^ ^,^ ^¶^	2%^§^ ^,^ ^¶^
Mt. Townsend, 2209 m	9.5 ± 3.1	40%^‡^	40%^‡^
Mt. Kosciuszko, 2228 m	8.6 ± 2.5	32%^‡^	49%^‡^

† The average maximum temperature over the period November 2009 to March 2010, measured using a temperature logger placed in a shaded place within the boulder field at each location (Green 2010a). Temperatures measured at Charlotte Pass from a Bureau of Meteorology Stevenson Screen over the same time period (12.3 ± 3.5°C) was not significantly different to that measured by the logger placed in the nearby boulder field.

‡ Data from Mountain pygmy possum (Gibson *et al.* 2018).

§ Data from Dusky antechinus.

¶ See Table 2, Note d.

**Fig. 3:**
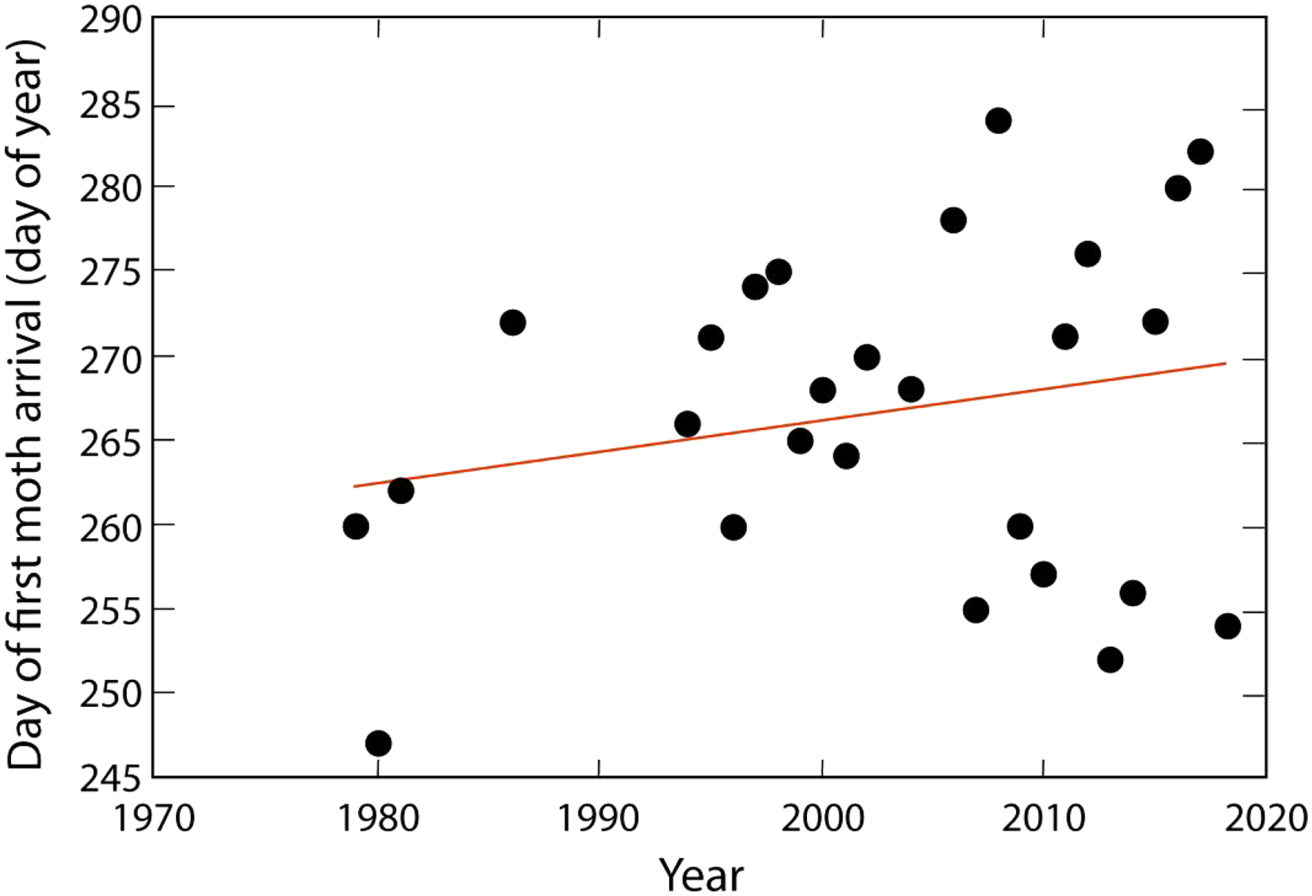
Date of first arrival of the Bogong moths above the snowline in the Snowy Mountains from 1979 until the present. *Regression line* has the following equation: *D* = 0.19*Y* – 114 (*R* ^2^ = 0.050) where *D* is the day of the year for the first moth arrival, and *Y* is the year.

The first arrivals of Bogong moths above the snowline have been recorded by one of us (KG) by ski surveys occurring on an almost daily basis from August until October since 1979 (Fig. 3). The earliest arrival ever recorded was in 1980 on 3 September. There is a considerable spread in arrival days across years, certainly due to yearly differences in weather conditions (e.g. harshness of winter). The latest arrival was recorded in 2008 – on 10 October – although for the years before and after, Bogong moths arrived almost a month earlier.

### Ascent and occupation of aestivation caves

When conditions become less favourable at lower elevations, for instance due to rising temperatures, Bogong moths ascend to their aestivation caves in the alpine zone above the tree‐line. Temperature is probably a major factor setting the lowest elevation where aestivation is possible, and this is likely to be related to summer temperatures within the caves, although significant variation in this temperature occurs depending on the location (Table 2).

**Table 2:** The lowest elevations (m) at which aestivation of Bogong moths takes place at different locations in the Australian Alps (with notes)

Location	Latitude	Lowest elevation (m)
Mt. Gingera, NSW/ACT	35.583°S	1760^†^
Bogong Mountains Big Plain/Mt. Jounama, NSW	35.576°S	1680/1710^‡^
Charlotte Pass, NSW	36.427°S	1755^§^
Unnamed peak ‘Ken Green Bogong’, NSW	36.517°S	1860^¶^
Mt. Tingaringy, NSW	36.998°S	<1440^††^
Mt. Higginbotham, Victoria	36.987°S	1720^‡‡^
Mt. Buffalo (The Horn), Victoria	36.736°S	1600^§§^

† The lowest ‘camp’ used as an aestivation cave was on nearby Little Ginini was at 1525–1585 m, but in 1952–1953, it was only used as a ‘temporary camp’ in spring and early summer (Common 1954, p229). Apart from that record, in current years, the lowest major aestivation site has been at 1760 m, below the peak of Mt. Gingera (which is at 1857 m).

‡ On 23 February 1999, Bogong moth remains were found in fox scats on the Bogong Peaks, and Bogong moths were seen flying around camp from about 7 pm (KG, pers. obs.). Moths flying at this time usually do so for approximately an hour before returning to the caves. Even though the reason for this flight is unknown, it may possibly be to drink, to gauge atmospheric conditions in preparation for their return migration, or for some other purpose. The location of the lowest cave used as an aestivation site is not known. At one site on Big Plain, Bogong moths were found on 26 January 2015 but not in large agglomerations (Ben Keaney, pers.com). The higher site on Mt. Jounama has not been examined.

§ At Charlotte Pass, the Mountain pygmy possum *Burramys parvus* was recorded with a diet consisting of 30% Bogong moths in spring, falling to ~1% in summer (Table 1, Gibson *et al.* 2018). This contrasted with higher elevation sites, where in summer the proportion of Bogong moths in the pygmy possum diet at Mount Townsend (2209 m) remained about the same as it was in Spring, while at Mount Kosciuszko the proportion increased throughout summer (Gibson *et al.* 2018). This suggests that Charlotte Pass was not a summer aestivation site and that the local lower limit for aestivation was thus at or above 1755 m.

¶ Bogong moths occurred in high numbers in pitfall traps and sheltered areas in the study sites of the Dusky antechinus *swainsonii* at 1860 m and 1940 m (PhD data, KG). This mainly occurred in spring over the 1981–1987 seasons. Lepidopterans, mainly Bogong moths*,* were the most common food through the spring in the diet of *A. swainsonii*, but this declined through summer (Green 1989). Frequency of occurrence of Bogong moths in scats of *A. swainsonii* was 75% in a high subalpine site in spring and 62.5% in a low alpine site (Green 1989). Numbers then appeared to decline over summer (Table 4 in Green 1989). However, this is a result of using ‘season’ rather than month in the published study, and when the data were re‐examined by month, the decline was found to occur in February with scats generally gone by March (PhD data, KG). In January at high subalpine sites, Bogong moth frequency of occurrence was 58.3% in *A. swainsonii* while in low alpine sites, it was 66.7% (PhD data, KG). In contrast, this had fallen to 11% and 2% respectively in February and 1% and 0%, respectively, in March, indicating that as summer became warmer (or moth numbers declined) the moths moved higher (PhD data, KG).

†† On 13 October 2001, Bogong moths were captured in their thousands while flying around the summit of Mt. Tingaringy. These moths smelled strongly of nectar and were being preyed upon by white‐throated needletails (a type of swift) as well as unidentified bats. On 15 October, moths were lined up inside a cave on the north‐west face of Mt. Tingaringy, with Quoll scats outside (which had been feeding on them). On 16 October ‘The Ampitheatre’ was found, a crack in the mountain estimated to be an 80 m length of accessible cave, with dead moths 8–10 cm deep on the floor (KG and Tony Stubbs). A big catch of moths was also collected on 5 and 6 January 2013, with a smaller catch on 7 Jan 2016 (KG, EW). Although many moths use the site, there is no record of occupation later than 7 January, so despite its size, there is currently no data to prove that Mt. Tingaringy is an aestivation site.

‡‡ Aestivation sites down to a lowest elevation of 1720 m (Ian Mansergh, pers. com.)

§§ For all summers from 1966–1967 and 1981–1982 (with the exception of 1968–1969), a cave (at 1600 m) on ‘The Horn’ on Mt. Buffalo was the lowest site used by Bogong moths to aestivate (MB).

### Numbers of aestivating moths 1951–2017: The decline

Qualitative observations of Bogong moth numbers at four main locations in the Australian Alps have been made continuously or sporadically by a number of individuals (including the authors) over the past 70 years (Table 3). A number of aestivation sites at each location were typically sampled. The locations were Mt. Gingera in the Brindabella Ranges, on the border of the ACT and NSW, Ken Green Bogong on the Main Range in the Snowy Mountains in NSW and Mt. Buffalo and Mt. Higginbotham in the Victorian alps.

**Table 3:** Records of Bogong moth occurrence and relative abundance at aestivation sites on four mountains in the Australian Alps^†^

Summer	Mt. Gingera	Ken Green Bogong	Mt. Buffalo	Mt. Higginbotham
1951–1952	1			
1952–1953	1			
1953–1954	1			
1954–1955	1			
1955–1956	1			
1956–1957	1			
1957–1958	1			
1958–1959	1			
1959–1960	1			
1960–1961	1			
1961–1962	1			
1962–1963	1			
1963–1964	1			
1964–1965	1			
1965–1966	1			
1966–1967	1		A	
1967–1968	1		A	
1968–1969	1		C	
1969–1970	1			
1970–1971	1			
1971–1972	1			
1972–1973	1			
1973–1974	1			
1974–1975	1		A	
1975–1976	1		A	
1976–1977	1	1	A	
1977–1978	1	1	A	
1978–1979	1	1	A^‡^	1
1979–1980	1	1	A^‡^	1
1980–1981		1	A	
1981–1982		1	A	
1982–1983		2	A or B^‡^	1
1983–1984			A or B	
1984–1985			A or B	
1985–1986			A or B	1
1986–1987		2	A or B	1
1987–1988		1	A or B	
1988–1989			A or B	
1989–1990	1		A or B	
1990–1991				
1991–1992			A or B	
1992–1993			A or B	
1993–1994		3	P	
1994–1995		3	P	
1995–1996		1	P	
1996–1997		2	P	
1997–1998		2	P	
1998–1999		2	P	
1999–2000			P	
2000–2001		1	P	
2001–2002		1	P	
2002–2003		2	P	
2003–2004			P	
2004–2005		1	P	
2005–2006		1	P	
2006–2007		3		
2007–2008		2	P	
2008–2009		3	P	
2009–2010		2	P	
2010–2011		1	A	
2011–2012	1	1	P	
2012–2013	1	2	P^††^	
2013–2014	1	2	A^‡^ ^,^ ^§^	1
2014–2015	3^‡‡^	1	A	3
2015–2016	3^‡‡^	1	C^§^ ^,^ ^¶^	3
2016–2017		1	C^§^ ^,^ ^¶^	3
2017–2018	4	4	C^§^ ^,^ ^¶^ ^,^ ^††^	4
2018–2019	4	4	4^§^	4

Numbers 1–4 refer to relative abundance of moths at their aestivation sites, as follows: (1) good, (2) normal to poor, (3) bad and (4) absent. For Mt. Buffalo, three cave sites were checked on one peak known as The Horn: two separated lower elevation sites (A and B at 1600 m), and several higher caves in close vicinity (C) ranging from 1680 m to the summit of The Horn at 1723 m – all sites may have been visited, but in certain years if site A had Bogong moths, the other sites (B and C) were not visited. A single letter indicates the lowest cave that moths were found in (e.g. C); A or B indicates that moths were found in one or both of these cave sites (and were thus also found at cave site C), but no exact record was kept. P indicates that moths were present in one or more of these cave sites, but no exact record was kept.

† Data were collected on (1) Mt. Gingera, NSW/ACT: 1952–1979 data from Common (1954, 1980) and pers. com., 1979 and 1989 KG, 2011–2014 EW, 2015–2019 PC, (2) the unnamed peak ‘Ken Green Bogong’, close to South Ramshead in the Kosciuszko Main Range, NSW: 1977–2019 KG, (3) granite boulder fields around Mt. Higginbotham, Victoria: data from Mansergh and Heinze (2019), (4) Mt. Buffalo, Victoria: 1966–2019 (as A, B and C) MB, with other Victorian records 1988–1989 Ian Mansergh – during 2013–2014 Dean Heinze reported that Bogong moth numbers were high, but did not appear to occupy either the low or medium cave sites.

‡ This was considered a good moth year on Mt. Buffalo.

§ Visit made in January.

¶ Bogong moths were only present in the summit cave on The Horn (1723 m)

†† A handful of Bogong moths were present.

‡‡ There is a possibility that Bogong moths were in good numbers during these years but did not occupy the caves on Mt. Gingera in order to take advantage of caves at higher elevations. Moths were in abundance from October until December but declined rapidly from the beginning of January (Caley and Welvaert 2018), that is, at least 1–2 months prior to the main return migration, possibly reflecting a move to higher elevations.

**Fig. 4:**
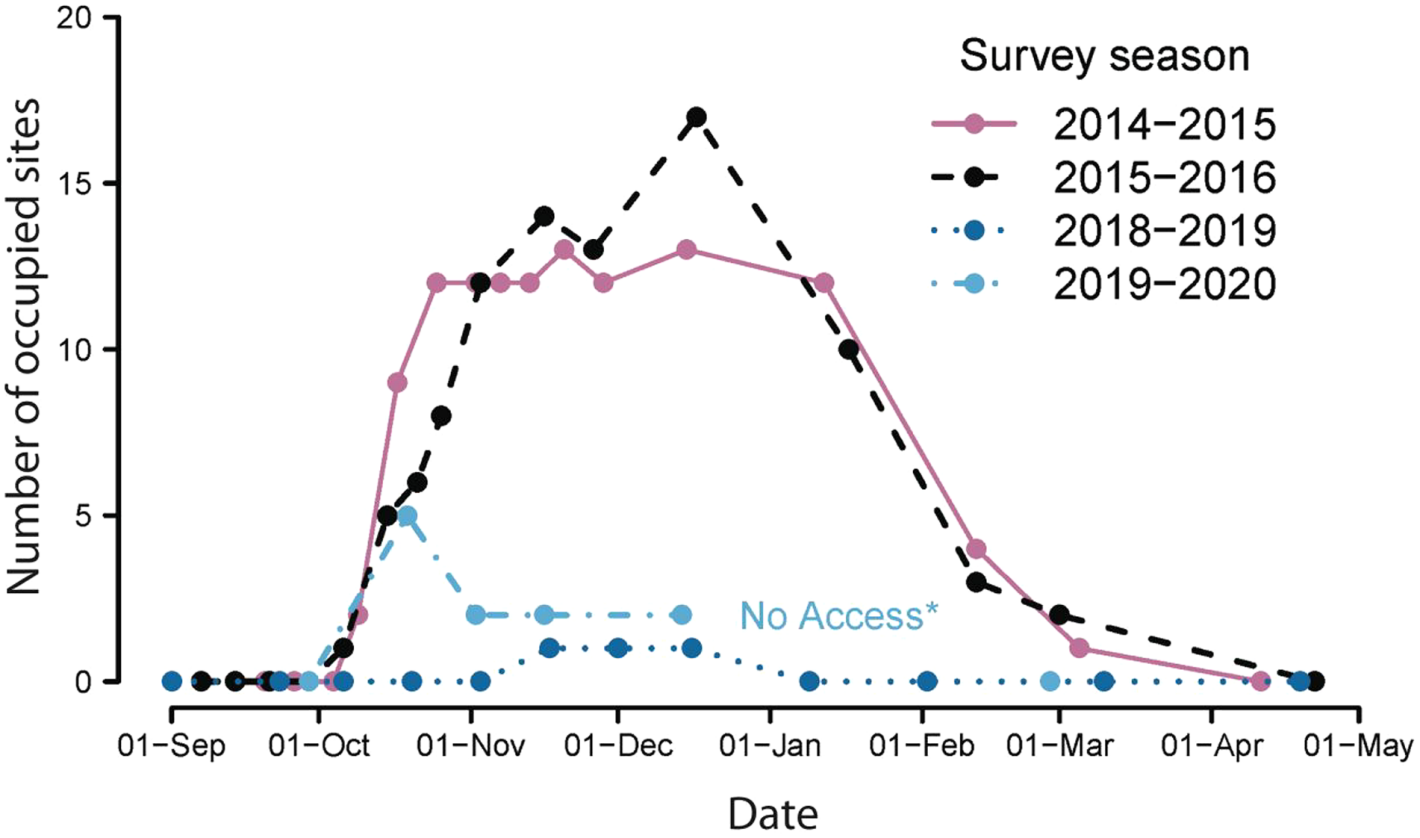
Seasonal changes in site occupancy of aestivating Bogong moths around the summit of Mount Gingera in the Brindabella Ranges (on the NSW and ACT border), based on 37 sites surveyed by Caley and Welvaert (2018) during 2014–2016 (reproduced with permission), and during the recent extreme drought.

Mt. Gingera is the site where Ian Common first described the aestivation and natural history of Bogong moths in the early 1950s (Common 1954). Common's counts revealed as many as 17 000 aestivating moths tiled onto every square metre of cave wall (subsequent estimates from the three‐dimensional spaces provided by a boulder field suggest >50 000 moths/m^3^: Mansergh and Heinze 2019). Common made at least one visit to Mt. Gingera every summer at the peak of the aestivation from 1951 until 1980, but made no mention of declining numbers over this period in his final paper on Bogong moths in the early 1980s (Common 1981), suggesting that moth numbers probably changed little. Records were not kept or available from Mt. Gingera from about 1980 until 2014. However, since 2014, one of us (PC) has made weekly or fortnightly surveys of 37 aestivation sites around the summit of Mt. Gingera. As mentioned above, during the summers of 2014–2015 and 2015–2016, Caley and Welvaert (2018) found that although Bogong moth numbers were high (Fig. 4), most had arrived, and also departed, 1 month earlier than observed by Common in the early 1950s (Common 1954). Their early arrival, as well as their early departure, long before the expected end of the complete aestivation period, suggests that moths were departing because conditions (possibly temperature and humidity) were no longer suitable for aestivation, a shift consistent with recent decades of climate warming.

Long‐term records of Bogong moth numbers from caves on Mt. Buffalo (1600–1723 m) in Victoria (started in the mid‐1960s) and caves at different elevations at Ken Green Bogong (1860–2020 m) in the Kosciuszko Main Range of NSW (started in the mid‐1970s), also reveal interesting trends (Table 3). At both locations, moth numbers were high and stable until the early 1980s. For all summers between 1966–1967 and 1981–1982 (with the exception of 1968–1969), Bogong moths were found regularly at the lowest site on ‘The Horn’ on Mt. Buffalo, at an elevation of around 1600 m, even during the drought period of 1979 to 1983, which revealed little impact on Bogong moth numbers either here or at Ken Green Bogong (Table 3). During the final summer of this drought (1982–1983), moths declined at Ken Green Bogong and possibly also at Mt. Buffalo (Table 3). This summer experienced the arrival of heavy deposits of red soil from drought‐impacted lands in the west of NSW (KG pers. obs., 20 November 1982).

From the early 1980s, moth numbers at Ken Green Bogong began to fluctuate significantly from year to year and on average began to decline (Table 3). Similar fluctuations were also noted within the basalt boulder fields of the Bogong and Hotham High Plains in Victoria's alpine region (Mansergh and Heinze 2019). At Mt. Buffalo, moths continued to occupy one or both of the two lower sites (at similar elevations) until at least the summer of 1992–1993; however, observations over the following 20 years do not reveal whether moths continued this trend or whether during some years they could only occupy the highest (coolest) caves.

### Numbers of aestivating moths 2017–2020: The crash

From the summer of 2017–2018, the numbers of aestivating Bogong moths we encountered in the Australian Alps fell dramatically (Table 3). In addition to the aestivation sites listed in Table 3, two others have also been regularly visited since 2013 (in conjunction with studies of the sensory basis of the Bogong moth migration, based at Lund University: Warrant *et al.* 2016; Dreyer *et al.* 2018): Mt. Morgan (1852 m) and Mt. Tingaringy (1434 m) in the northern and eastern Snowy Mountains, respectively.

In the summer of 2017–2018, moths were absent from Mt. Morgan and Mt. Gingera (observations were made on 7 and 8 January, respectively, and again on 4 and 2 February, respectively). At the summit of Ken Green Bogong, which was visited on 10 January and 5 February 2018, the walls of the main cave were empty, with moths only found in deep crevices. This was the lowest number of Bogong moths ever recorded for this location (KG), with a similar report from Mount Buffalo for 18 and 19 January 2018 (MB).

During the following summer season of 2018–2019, Mt. Buffalo was investigated on 16 December 2018, and no moths were found (KG). The following day, moths were observed in three deep locations of the major upper cave on Ken Green Bogong (2020 m). Three other caves at lower elevations near Ken Green Bogong were empty, apart from three moths inside one cave (KG). At the same time on Mt. Gingera, very few moths were observed (Fig. 4). On 7 January 2019, only one live Bogong moth was found on Mt. Morgan, and three were found on Mt. Gingera. On 9 January, seven moths were observed at Ken Green Bogong. At higher elevations on the Kosciuszko Main Range, larger numbers of moths were however found at two locations (KG): Club Lake (1950 m) on 10 February and Mt. Kosciuszko (2100 m) on 21 February 2019. Further south in Victoria, in early January 2018, the only Bogong moths observed on Mt. Buffalo were a handful of moths in the upper cave under ‘The Horn’ (1723 m), while in January the following year (2019) moths were entirely absent (MB). This almost complete absence of Bogong moths in previously richly populated caves, both in NSW and Victoria, had never previously been observed in over half a century of observations.

During the spring of 2019, unexpectedly high numbers of Bogong moths flew into the Snowy Mountains and the Victorian Alps, despite little relief in the drought. Why or how this was possible is not clear. There were localised heavy falls of rainfall in areas of southwest Queensland from February to April 2019, leading to flooding in some areas of southern Queensland that extended into northern NSW. These weather patterns also led to reasonable falls in localised areas of western NSW that may have promoted Bogong moth reproduction and survival. However, no matter what the cause, the increased numbers of moths entering the mountains during the spring of 2019 translated into considerably larger aestivating cave populations than seen in the previous two summers (but nowhere near as high as before the drought), although apparently only at higher elevations. On Mt. Gingera (Fig. 4), a lower elevation site, the number of aestivation sites occupied were many fewer than during previous recent summers (with the exception of the 2018–2019 summer when the numbers of occupied sites were even fewer). Following the devastating subalpine fires of early January 2020, few Bogong moths remained at these high elevation aestivation sites after mid‐February, either having succumbed to smoke, or having been washed out of the caves by heavy rains in the first half of February or having departed for the breeding grounds earlier than normal.

## Discussion

Our results, taken together with observations by Ian Common in the early 1950s, suggest that the Bogong moth population was stable until the early 1980s but since then has fluctuated, and on average slowly declined, until crashing over the last 2–3 years. This rapid decline has probably had a deleterious impact on the health of the alpine ecosystems of south‐eastern Australia which depend on the massive influx of energy and nutrients that Bogong moths provide via their summer migration to the mountains (4929 GJ of energy, 7.2 tons of nitrogen and 0.97 tons of phosphorus: Green 2011).

The years 1951–1980 can probably be considered a baseline for ‘normal’ post‐European population numbers of Bogong moths (Common 1954, 1981). In winter 1980, there was an extensive outbreak of caterpillars in south‐western NSW, with Bogong moths being particularly numerous (Drake and Farrow 1985). This winter outbreak also coincided with the earliest records of Bogong moth arrivals above the snowline of the Snowy Mountains (Fig. 3), and that summer moth numbers were not only high in the Snowy Mountains but also in the Victorian Alps, at Mt. Buffalo (Mansergh pers. com. 2019). Moth numbers subsequently began to decline in Victoria (Table 3) although there is no data for Mt. Gingera (Ian Common last wrote of Bogong moths on Mt. Gingera in 1981), nor for the Snowy Mountains (between 1983 and 1994, only one record is available: from 1988 – KG pers. obs.). From the mid‐1990s, moth numbers started to fall in the Snowy Mountains and likely also on Mt. Buffalo (Table 3).

Admittedly, the data we present here are necessarily sketchy. Until very recently, data on moth numbers were collected either for scientific reasons (to answer completely different questions from those presented here), or as a result of curiosity and interest (e.g. the long‐term records of MB from Mt. Buffalo). In the past, few could have predicted that Bogong moths, usually common and plentiful in the Australian Alps, could now become so threatened, thus motivating the type of extensive population monitoring that would have strengthened the conclusions of this review (and which are likely warranted in the future). Thus, our data only represent a handful of aestivation sites – fewer than 70 – although these are spread across their full latitudinal range and at a number of elevations. But this admittedly fragmented data – complemented by analyses of the faeces of Bogong moth predators – show that Bogong moth numbers are worryingly in decline.

There are several possible causes of this decline, including agricultural practices (e.g. habitat loss and insecticides), climate change (particularly drought) and other factors (e.g. foxes, pigs, traditional hunting and urban light pollution). These factors have been implicated in the recent dramatic declines of insects across the globe, both in terms of population loss and species extinctions (with Lepidoptera prominent: Hallmann *et al.* 2017; Sánchez‐Bayo and Wyckhuys 2019; Goulson 2019; Wagner 2020). We discuss each of these factors in turn.

### Agricultural practices

Agricultural practices in the landscape are very likely to have had an impact on Bogong moths. Unfortunately, there is no consistent data on whether Bogong moth numbers have declined in the breeding areas of western NSW (and hence in the mountains). If, after 1980, farming practices gradually reduced the number of Bogong moths in the breeding areas over the following 35 years, it is still difficult to disentangle agricultural impacts (e.g. habitat loss and pesticide use) from impacts arising from the increasing temperatures that have resulted from climate change.

To address the possible impacts of pesticides, Bogong moths were collected in 2001 from a cave at Ken Green Bogong (1940 m) and were analysed by the NSW Department of Agriculture for sublethal levels of 59 chemicals currently used in agriculture. These analyses revealed no traces of these agricultural chemicals. Bogong moths killed by spraying in Parliament House in Canberra were collected in spring 2003 and were analysed for 139 different chemicals by the Australian Environmental Protection Authority. The pesticide used – Deltamethrin (USEPA/OPP Pesticide Code: 097805) – was recorded at a rate of 0.95 mg/kg. Deltamethrin is a pyrethroid ester insecticide and the main constituent of Cislin 10, one of the most popular insecticides in the world. Apart from Deltamethrin; the moths collected at Parliament House contained only trace concentrations of other pesticides (Hansard 2003).

When deltamethrin enters the soil, it binds tightly to soil particles (National Pesticide Information Centre 2010). Deltamethrin is commonly used against Lepidoptera and kills on contact, or by larval consumption in the soil (EXTOXNET 1996). It does not persist in soil, with degradation occurring within 1–2 weeks (EXTOXNET 1995). This suggests that Deltamethrin would generally kill Bogong moths. The recent use of neonicotinoids (which has a persistence in plants of 1–2 years) has also very likely impacted the survival rate of Bogong moth larvae in the breeding areas where applied. New chemical analyses of Bogong moths are urgently needed to check whether neonicotinoids are present in their tissues.

Chemical spraying of pesticides at Bogong moth breeding sites occurs mainly on broadacre farmland in areas of grain production that lie in the central‐east and southern regions of NSW and extend into southern Queensland (Fig. 1). The biggest farming change in this area has been the southward shift of cotton growing, taking in large areas along the Murrumbidgee River (Michael Nash, pers. com.). The pesticides now used (and their quantities: Zalucki *et al.* 2009) may well have changed since Bogong moths were collected and analysed in 2001 and 2003 (Green 2008). Indeed, the introduction of neonicotinoids in the late 1990s to broadacre farming regions of southeast Australia (Nash *et al.* 2019) may now be having an impact on Bogong moth populations. These insecticides are currently under heavy scrutiny for their adverse effects on economically beneficial insects such as bees and were partially banned in Europe in 2013. Even though the specific effects of neonicotinoids on lepidopterans is currently unknown, various species of moths (and particularly noctuids) are considered agricultural pests and are no doubt a target of these insecticides.

Little insecticide was used on arable dryland country in the Murray‐Darling Basin (Fig. 1) over the ‘last couple of years’ due to drought conditions (Michael Nash, pers. com.). Further west, in rough grazing and native vegetation country (Fig. 1), traditional pastoral lease holders would have used even lower amounts of pesticides. Thus, the extent to which neonicotinoids and other types of insecticides may have contributed to the Bogong moth decline is presently unknown.

Two agriculture activities made possible by the availability of irrigation water – the production of rice and cotton – may also adversely affect Bogong moth numbers. In Australia, rice is grown in irrigated fields which exclude Bogong moths (since the larval and pupal stages are subterranean). On average, the annual production area of rice is between 65 000–90 000 ha (most of which is in the Murrumbidgee and Murray valleys – Plant Health Australia 2020). Since Bogong moth density in these areas is estimated at 0.78 moths per 10 m^2^ (Green 2008, see above), the loss of moths due to habitat loss would be 50.7–70.2 million moths annually, compared to pre‐European conditions.

Cotton plantings may have also impacted Bogong moth numbers. Disregarding any impacts by spraying, which has decreased since the 1996 introduction of genetically modified cotton (which expresses a *bt* toxin gene: Zalucki *et al.* 2009, Zalucki 2015), cotton fields are monocultures without any grass between the cotton plants – hence resulting in no suitable food plants for Bogong moth larvae. Although some cotton has been grown in Australia since the 1820s, by 1951, an established cotton industry was still essentially non‐existent. Using water from newly constructed dams, cotton production increased to 435 000 bales per annum by 1980, and by 1985, it had more than doubled to 1.1 million bales per annum. A further doubling occurred by 1992, and by 2011–2012, production was 5.3 million bales per annum (de Garis 2013). Even though the combined area of cotton fields that have successfully produced cotton has fluctuated significantly over the last two decades due to drought (Zalucki 2015), the area of cotton fields themselves (productive or otherwise) has grown. For Bogong moth areas in NSW and southern Queensland cotton fields cover an area of 483 890 ha. Whether productive or not, these cotton fields remain fallow and empty over winter and are thus highly unsuitable as Bogong moth breeding grounds. Again, assuming 0.78 Bogong moths per 10 m^2^, and assuming cotton growing areas do not support Bogong moths, this means the loss of a further 377.4 million moths annually, compared to pre‐European conditions.

Thus, cotton and rice cultivation in the grey cracking clays natal areas has resulted in a current‐day reduction of almost half a billion moths from the Bogong moth population annually. Of these, around half would likely have arrived in the mountains (where close to four billion are thought to arrive annually in regular years: Green 2010b, and see above). This loss – currently around 1/16 of total moth arrivals – gradually grew from 1980.

Thus, habitat loss via rice and cotton farming have almost certainly contributed to the slow long‐term decline of the Bogong moth. The role of synthetic pesticides is much less clear, and longer‐term monitoring studies, particularly to determine the impact of neonicotinoids and other commonly used insecticides on Bogong moth decline, are urgently required to quantify these roles more precisely.

### Climate change

#### Effects of temperature

##### Arrival of moths in the mountains

Increasing temperatures through climate change appears to have had no impact on the arrival date of Bogong moths in the Snowy Mountains as arrivals have been highly variable (Fig. 3). However, the arrival date of Bogong moths does have an impact on alpine fauna because for many species they provide the only available food immediately after the snowmelt. Animals dependent upon winter snow cover are becoming active earlier because of a decreasing snow mass and an increasingly earlier snowmelt (on average, about 2.75 days earlier per decade since the early 1950s: Green 2010a). With little else as a food source, an earlier arrival date for Bogong moths thus becomes critical. However, there is no evidence for this as arrival dates are highly variable (Fig. 3, Green 2010a). For instance in 1952, a good moth year, Bogong moths were recorded arriving in the Brindabella Ranges in early September, nearly a month earlier than in 1951 (Common 1954). A similar difference in first arrival dates (37 days, from 4 September to 11 October) occurred in the Snowy Mountains during 40 years of observations (Fig. 3). As with Common's 1952 record, the earliest arrival date recorded in these later observations (Fig. 3) was in 1980, which was also a ‘good’ moth year. However, an early arrival date is not necessarily associated with a good moth year – the early arrival years of 2007 and 2009 were years with low numbers of moths, as appears to be the case for the third earliest arrival in 2019, while the late arrival years of 2012 and 2016 were years with high numbers of moths. These observations suggest that some other factor, rather than the snow duration in the mountains, determines either the arrival date in the mountains or the date of departure from the breeding areas. Indeed, factors along the migratory route – such as weather conditions or the availability of food from flowering plants such as eucalypts – may affect the length and/or duration of the migratory journey and thus the arrival time in the mountains.

##### Aestivation

Despite differences in arrival date, the movement of Bogong moths into the aestivation caves is more likely to be affected by the climate in the mountains themselves. In both 1951 and 1952 on Mt. Gingera, Common (1954) first recorded Bogong moths in the ‘chief observational cave’ (an aestivation cave) on 20 November in 1951 and 26 November in 1952. However, the arrival of moths in their aestivation sites in 2014 and 2015 was approximately 6 weeks earlier (Fig. 4, Caley and Welvaert 2018). Moreover, Caley and Welvaert (2018) noted that the greatest numbers of moths occurred on 1 November 2014 and 5 November 2015 and that moth numbers had already declined before the date that Common recorded the maximum moth number in the same cave in 1951 and 1952 (1 January). This suggests that Mt. Gingera is no longer a preferred aestivation site, but rather has become a temporary camp before ascent to caves at higher elevations. Indeed, even as early as 1954, Common had hypothesised that Bogong moths might use Mt. Gingera as merely a stopover during their migration route between it and higher elevations in the Snowy Mountains, both in the autumnal migration and possibly in the summer, as in the hot summer of January to February 1952 (Table 4).

**Table 4:** Average maximum temperatures (°C) in January and February during different years from caves in the NSW and ACT Alps

Location	Year	January	February
Mt. Gingera NSW/ACT^†^ 1840 m	1952	14.5	12.3
Mt. Gingera NSW/ACT^†^ 1840 m	1953	10.5	9.5
Mt. Gingera NSW/ACT^†^1840 m	2020	16.2	13.1
Charlotte Pass NSW 1755 m	2010	13.8	12.2
Unnamed peak ‘Ken Green Bogong’ NSW 1940 m	2006	13.7	12.3
•	2007	12.1	12.0
•	2008	12.8	8.5
•	2009	12.3	11.3
•	2010	12.4	11.1
•	2014	12.5	12.7
•	2015	10.5	11.8
•	2016	10.8	12.1
•	2017	13.4	11.4
•	2018	12.8	11.2
•	2019^‡^	15.7	11.4
Unnamed peak ‘Ken Green Bogong’ NSW 1940 m	2020	13.3	10.9

† Common 1954. The cave monitored by Common on Mt. Gingera was at about 1840 m, just below the summit at 1857 m. The site monitored in 2020 (35.5765°S, 148.7790°E) is a deep narrow crevice within a jumble of boulders approximately 50 m from, and at a similar elevation to, Common's cave. This site was chosen as it has been the most reliable site for over‐summer aestivation since 2015.

‡ During the latter half of December 2019, the average maximum temperature reached 15.6°C.

The fact that moth declines are becoming noticeable at lower elevations, such as those close to the summit of Mt. Gingera (ca. 1840 m), can be explained by either of two possible (nonmutually exclusive) scenarios: (1) lower elevation aestivation sites are becoming less suitable due to climate change or (2) higher elevation aestivation sites have always been preferred by the moths, but when high numbers of moths filled the higher sites, additional moths were forced to aestivate at the lower (and less‐preferred) sites, so that when moth numbers began to decline, these lower sites emptied first.

Both of these scenarios are supported by the long‐term moth declines observed at lower elevations in the Australian Alps. The lower cave on Ken Green Bogong has almost always had a high number of moths in early spring–summer, but numbers decline to zero in mid‐summer as moths move higher. This situation could well have been mirrored on Mt. Gingera during the summers of 2014–2015, 2015–2016 and 2019–2020 when Bogong moth numbers first rose and then declined (up to 2 months earlier than they did during the summer of 1953–1954: Common 1954; Caley and Welvaert 2018; Fig. 4). Based on the January 2019 mean maximum temperature of 15.7°C in the lower (1860 m) cave at Ken Green Bogong (Table 4), the mean maximum temperature of the upper cave (2020 m) would have been 15.0°C (Green 2014). This temperature, higher than that measured by Common (1954) in the cave near the summit of Mt. Gingera (ca. 1840 m) during the hot summer of 1951–1952, suggests that Ken Green Bogong may have been unsuitable for Bogong moths and that they might have migrated further up hill. Indeed, during the summer of 2018–2019, Bogong moths appeared to be in good numbers only in locations just below the summit of Mt. Kosciuszko where cave temperatures would have been around 13.0°C (Green 2014). Thus, during this summer, the upper cave at Ken Green Bogong was either too warm (at 15.0°C) for aestivation (Scenario 1), and/or the lower numbers of moths in the mountains were entirely accommodated within the highest (and preferred) aestivation sites, leaving the lower sites (like those at Ken Green Bogong) empty (Scenario 2).

The first scenario – that lower aestivation sites are becoming less suitable due to climate change – would require that these sites have acquired average maximum temperatures that exceed the maximum for which aestivation is permitted. However, this maximum temperature has never been determined scientifically.

What might this maximum temperature (and lowest elevation) be? The lowest definitely known and regularly frequented whole‐summer aestivation elevation is 1600 m at Mt. Buffalo – equivalent to 1700 m at Mt. Gingera when one accounts for Mt. Buffalo's 1.15° more southerly latitude. This is only slightly lower than the lowest known aestivation sites below the summit of Mt. Gingera (which are located at 1760 m: Table 2). In the northern Snowy Mountains, alpine pygmy possums have been recorded eating Bogong moths in January at Boltons Hill (1581 m, above Happy Jacks Valley; Linda Broome, pers. comm.), suggesting whole‐summer aestivation at that location. In contrast, almost 100 m lower at nearby Snow Ridge (1495 m), pygmy possums are found to consume Bogong moths almost exclusively in Spring (as revealed by scat analyses in 2011: Gibson *et al.* 2018) – presumably the moths do not remain here but instead migrate to higher elevations in the early summer. In the eastern Snowy Mountains, moths have also been observed in good numbers until at least 2015 at Mt. Tingaringy (1440 m), although it is not certain that they are there for the entire summer (Table 2, note e), or if they move to higher elevations in January (the more likely scenario).

Thus, the lowest elevation for regularly frequented whole‐summer Bogong moth aestivation sites is likely to be around 1600 m, both in NSW (Boltons Hill) and Victoria (Mt. Buffalo). What do these elevations suggest about the maximum permissible aestivation temperature? Data given in Table 4 reveal that between 2006 and 2020, the average maximum temperature measured from a cave at Ken Green Bogong (1940 m) during January was 12.7°C. Using a lapse rate for the Snowy Mountains of 9.1°C per kilometre of elevation (Green 2014), one can calculate that during the same period the average maximum temperature from the lowest suspected aestivation site on Boltons Hill (1581 m) would have been 16.0°C. We suggest that this is likely to be close to the highest temperature that permits aestivation. Supporting this notion is an unusual observation made by Ian Common during the summer of 1951–1952 on Mt. Little Ginini in the ACT (1525 m, and close to Mt. Gingera). Common noted that Bogong moths aestivated on Mt. Little Ginini for the entire summer when the average January to February maximum temperature was projected to be 14.8°C (Table 5; Common 1954). This suggests that aestivation is permitted at temperatures at least as high as 14.8°C, only 1.2°C lower than our predicted maximum. Thus, 16°C seems to be a reasonable estimate for the maximum temperature that permits aestivation.

**Table 5:** Mean daily maximum temperature *T* from January to February at aestivation sites on Mt. Gingera^†^ and Mt. Little Ginini (1952–1953)^†^, in the Snowy Mountains (2010), and on Mt. Buffalo (1967)

Location	Elevation (m)	Mean T (°C)
Mt. Gingera 1952	1840	12.7^‡^
Mt. Gingera 1953	1840	10.6
Mt. Gingera 2020	1840	14.7
Mt. Little Ginini 1952	1525	14.8^§^
Mt. Little Ginini 1953	1525	12.7^§^
Charlotte Pass	1755	13.8
Unnamed peak ‘Ken Green Bogong’	1860	11.8
Unnamed peak ‘Ken Green Bogong’	1940	11.9
Unnamed peak ‘Ken Green Bogong’	2020	11.5
Mt. Ramshead North	2140	11.7
Mt. Twynam	2150	8.9
Mt. Townsend	2150	10.4
Mt. Kosciuszko	2200	10.2
Mt. Buffalo (Vic)	1600	13.2

† Common 1954. The cave monitored by Common on Mt. Gingera was at about 1840 m, just below the summit at 1857 m. The site monitored in 2020 (35.5765°S, 148.7790°E) is a deep narrow crevice within a jumble of boulders approximately 50 m from, and at a similar elevation to, Common's cave. This site was chosen as it has been the most reliable site for over‐summer aestivation since 2015.

‡ In 1952, it appears that temperatures were measured in a location that may not have been fully shaded (Common 1954, p254). If we assume that January temperatures did not exceed those at Kiandra, and set Mt. Gingera's January temperature at 13.0°C, the corrected 1952 January to February mean temperature would then be 12.7°C.

§ Extrapolated from temperatures measured at the summit of Mt. Gingera.

From the turn of this century, Bogong moths at aestivation sites on Mt. Gingera have declined during a 0.7°C temperature increase (i.e. 0.36°C per decade: Sánchez‐Bayo and Green 2013). The mean maximum January temperature in the currently preferred aestivation site (at around 1840 m) was 16.2°C during 2020 (Table 4) – at or above our estimated maximum permissible temperature for aestivation. These aestivation sites, together with those on Mt. Buffalo, are among the lowest aestivation sites known. There is no question that if climate change advances and global temperatures continue to rise, aestivation will become more and more restricted to sites at higher elevations. To determine the actual maximum temperature where aestivation is permitted, the temperatures of caves at different elevations where moths aestivate need to be monitored using temperature loggers. Such measurements will allow us to forecast the impact of climate change on the Bogong moth population in the mountains and allow us to predict the impacts that changes in this population will have on the alpine ecosystem.

#### Effects of drought

From 2017 to 2019 much of the agricultural lands of the Murray–Darling Basin in western NSW and southern Queensland were affected by extreme drought, the most severe in the recorded history of the Murray–Darling Basin. This drought has had a major impact on the Bogong moth population, whose main breeding areas occur in this region. The Bogong moth crash seen in the Australian Alps over the summer of 2017–2018 was sudden, widespread across all mountain areas, and continued into the summers of 2018–2019 and to a lesser extent 2019–2020.

**Fig. 5:**
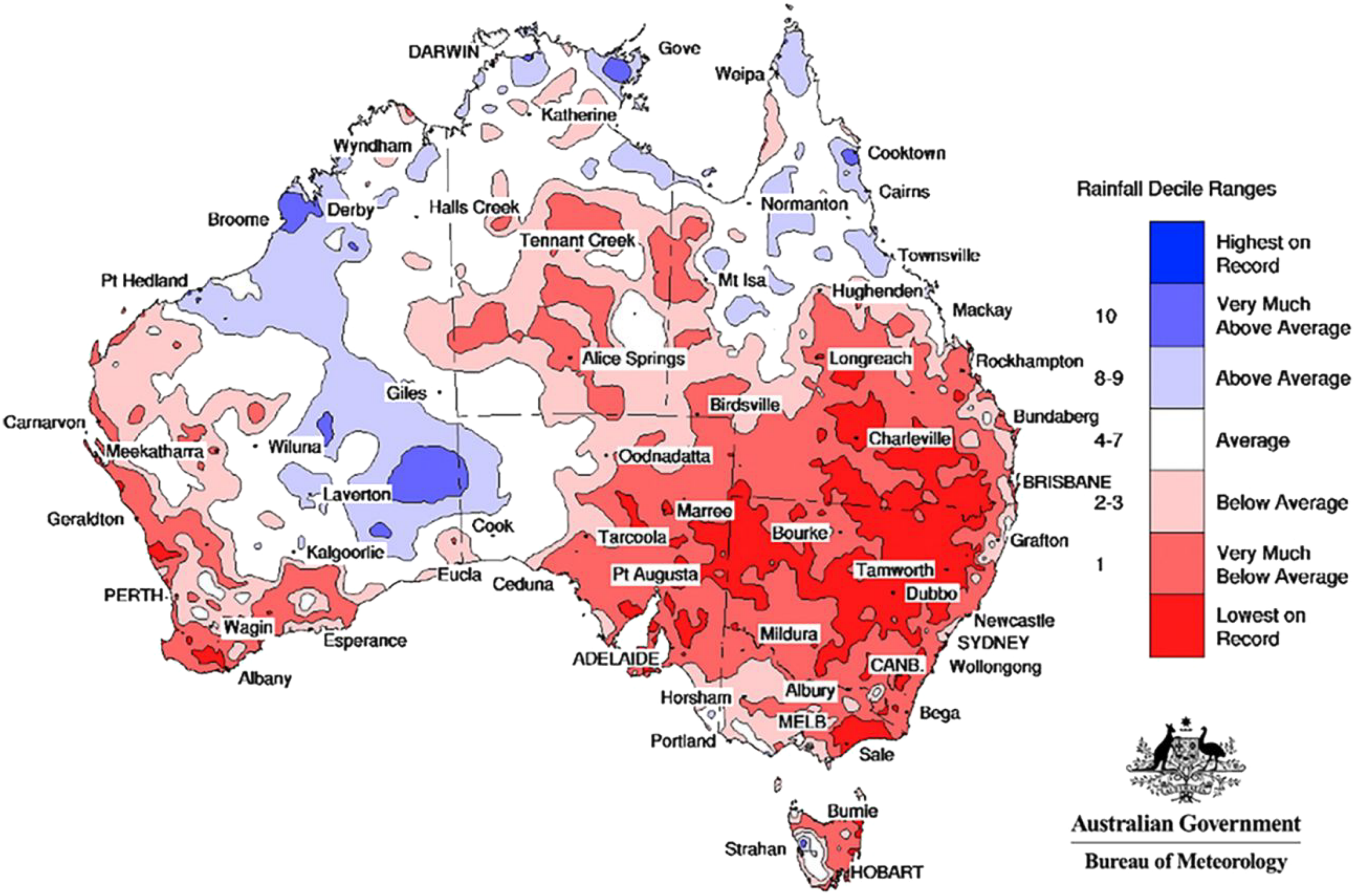
Australian rainfall deciles (1 January 2017 to 31 December 2019). Source: Bureau of Meteorology, Australian Government. Distribution based on gridded data.

There is no doubt that the major cause of the recent Bogong moth crash is the latest drought, which may have affected moths in a number of ways. Firstly, a lack of food for the larvae in the breeding areas would have been a major problem, coupled with unfavourable soil temperatures and moisture levels. We do not know the direct effect of temperature on the larvae, but temperatures were higher than normal, and coupled with a considerably drier soil and a lack of food, there were probably very few larvae that survived the dry winter of 2017, and thus very few adults that emerged and flew to the mountains in spring. Of course, even fewer of those that migrated to the caves would have survived to return in autumn for the winter breeding season of 2018. Those few that did return would have encountered the same drought‐stricken landscape that they had experienced the previous year, leading to even fewer of their offspring surviving to adulthood. Because the winters of 2018 and 2019 were equally dry (Fig. 5), it is not difficult to understand how Bogong moth numbers plummeted.

The effect of drought on Bogong moths numbers prior to 2017 is less clear, although significant fluctuations are apparent (as has also been noted in Victorian alpine areas: Mansergh and Heinze 2019). Almost all of eastern Australia was affected by major droughts in 1958–1968 and 1979–1983, with the 1982–1983 El Niño that developed from June to August creating severe conditions in terms of lack of rainfall and extreme heat. There was no noticeable impact of these droughts on Bogong moth numbers at Mt. Buffalo apart from the significant temporary reduction experienced in summer 1968–1969. However, by the summer of 1969–1970, Bogong moth numbers were back to ‘normal’ (Table 3). In the summer of 1972–1973, the Bogong Peaks were reported as the only site in NSW with Bogong moths in any quantity (Flood 1980). However, it is not clear whether any other major aestivation sites in NSW were checked by Flood (1980 – Blakers 1980). Initially, the drought from 1979 to 1982 made no difference to Bogong moth numbers on Mt. Buffalo, in the Snowy Mountains or during the one summer recorded at Mt. Gingera. Moth numbers in the Snowy Mountains and on Mt. Buffalo declined in the summer of 1982–1983 and remained lower at Mt. Buffalo, but there are no records available elsewhere.

The Millennium Drought in southeast Australia (2001–2009) was particularly severe and badly affected the Murray‐Darling Basin and virtually all of the southern cropping zones (Australian Bureau of Meteorology 2015). Bogong moths continued to be found at lower levels on Mt. Buffalo but the drought had major impacts on moth numbers in the Snowy Mountains during the summers of 2006–2007 and 2008–2009 (Table 3). However, the successive La Niña events during the summers of 2010–2011 and 2011–2012 were associated with record rainfall over much of Australia (Australian Bureau of Meteorology) and resulted in good years for Bogong moth numbers in the Australian Alps. At this point, it was obvious that there had been a long‐term decline in overall numbers of Bogong moths, with declines that followed the drought of 1981–1983 and further declines that commenced in 1993–1995 that pre‐empted the Millennium drought (Table 3). The decline in numbers from 2014 to 2015 on Mt. Higginbotham, coupled to the progressive movement of Bogong moths to higher elevations, suggest that when numbers are low, Bogong moths on Mt. Buffalo move to higher, cooler locations. This also appears to be occurring further north in NSW on Mt. Gingera (35.58°S), where the lowest aestivation sites lie at 1760 m and continue upwards to just below the summit at 1857 m. This is essentially the same elevation band for aestivation sites a little further south at Charlotte Pass (36.43°S): 1755 to 1837 m. Bogong moths found here are generally spring arrivals that migrate to higher elevations as summer develops (see above). The latest drought began in NSW in mid‐2017, with Bogong moth numbers crashing within the breeding areas. Since then, there was no reprieve in the drought until well in to 2020 – NSW was declared to be 100% in drought by August 2018 and Queensland 65.2% by May 2019 (NSW Department of Primary Industries 2019). By July 2019, the drought was officially recorded as the worst on record in the Murray–Darling Basin (Farm Online National 2019), exceeding the Millennium drought, Federation Drought (1895–1903) and the World War II drought (1937–45). By the end of March 2019, rainfall levels in the Murray–Darling Basin were the third lowest on record. Temperatures were extremely high, with the nearest equivalent temperatures, according to paleoclimatic data, being a hot period that occurred two to three million years BP (Dr David Jones: Australian Bureau of Meteorology). Together, these conditions suggest that the recent drought, mediated by climate change, is sufficient in itself to have caused the Bogong moth collapse.

### Other factors

In addition to agricultural practices and climate change, annual declines of Bogong moth numbers may have occurred for the following reasons: (1) attrition as larvae in soil that is too dry and/or too warm (as likely occurred during winter 2017 and 2018), or attrition during adult migration as a result of (2) introduced predators, (3) environmental factors (such as increasing light pollution) or (4) because of attrition once the moths reach the mountains (e.g. due to climatic factors, and introduced predators and parasites). Obtaining a measure of the numbers of moths existing as larvae (Green 2008), or as adults during migration, is difficult, particularly if relying on light traps. Local and synoptic weather systems can both affect the moth catch in light traps due to thunderstorms and the passage of cold fronts or depressions. The wind speed, air temperature and levels of moonlight may also have an affect (Gregg *et al.* 1994). The aestivating population of Bogong moths, from their arrival at their caves in December until the beginning of their departure in early March, is the part of the lifecycle where numbers of moths can be most accurately measured.

Ten years ago, a total number of 2.2 billion aestivating Bogong moths was estimated to exist in the Snowy Mountains over the summer (Green 2011). This number suggested that observed losses of moths in cities such as Canberra – where in occasional years migrating moths have been lured by city lights after presumably being blown off course by unusually strong winds – may not have had an overwhelming impact on overall numbers in the mountains as most moths eventually leave their city refuges and continue their journeys. These losses from light pollution were probably comparatively small (although the loss of moths by any cause is now becoming important). Apart from light pollution, another major change since European settlement is the arrival of feral predators such as foxes (arriving in the Snowy Mountains around 1900, after the first capture of a fox in NSW in 1893: Rolls 1969). Fox predation now accounts for a loss of 55 imperial tons of Bogong moths annually in the Snowy Mountains (Green 2011). However, fox baiting from 1999 onwards (which still continues annually above the treeline) had an immediate impact on fox numbers, with a decline of about 80% based on three criteria: fox scat counts, fox tracks found on snow transects, and numbers of foxes counted at Charlotte Pass (K. Green, unpublished data). A recent report indicates that wild pigs have also become significant predators of Bogong moths, following the introduction of these feral animals to the Snowy Mountains in 1959 (Caley and Welvaert 2018). Before European settlement, a major impact on Bogong moths was hunting by Aboriginal people, and there are estimates that this would have taken several tons of moths (Jardine 1901). However, without the actual figures, the impact of these hunting activities on moth numbers cannot be calculated.

## Conclusions

Records of Bogong moths collected over the past 70 years from around 50 to 60 aestivation sites, and more recently along the migratory route, coupled with the analysis of Bogong moth remains in mammalian predator faeces, reveal noticeable patterns in the numbers of moths present over summer in the Australian Alps. Even though the data (our own and those of others) are admittedly fragmentary, this analysis has revealed that Bogong moth numbers were fairly stable from 1950 until around 1980, apart from temporary falls in numbers due to occasional drought. From 1980 until 2016, Bogong moth numbers in the mountains began to fluctuate, with some years having very high numbers of moths, while others had many fewer. Even though overall numbers remained high, these years showed evidence of a slow decline. From 2017 to 2020 a sudden and worrying crash in Bogong moth numbers occurred.

Although the causes for the decline and recent crash in Bogong moth numbers remain uncertain, one or more factors may be responsible. We currently have little evidence to suggest that increasing global temperatures *per se* are responsible for the decline in Bogong moths seen across the mountains; however, the Australian climate has nonetheless become continuously drier and warmer over past decades. This change in climate has possibly had a serious impact on the ability of Bogong moth eggs, larvae and pupae to survive the winter in their breeding grounds, which in turn may have slowly been reducing the number of moths entering the mountains. Moreover, our analysis also suggests that the maximum temperature for aestivation may be around 16°C, and that a warming climate is thus likely restricting aestivation to higher and higher elevations. These possible effects of climate change, added to known changes in farming practices, such as increasing land clearing for crops and an elevated use of insecticides, may account for the gradual decline in moth numbers observed from about 1980 until 2016. These declines were followed by the catastrophic Bogong moth summers of 2017–2018 and 2018–2019 (and to a lesser extent 2019–2020) for which only drought can be seen as the cause. This drought is very likely to have severely impacted the survival of Bogong moth immature stages in their breeding grounds, a speculation supported by the sudden disappearance of Bogong moths that we observed along their migratory routes during Spring. The latest drought – the worst in recorded history in the Murray–Darling Basin – is almost certainly the result of climate change (CSIRO 2018).

Finally, as with the alarming worldwide decline of insects in general (Wagner 2020), the decline of Bogong moths in the Australian Alps should be seen as a worrying development. The delicate alpine environment is already severely threatened by climate change, and the massive annual influx of energy and nutrients provided by Bogong moths is of major importance for the health of this ecosystem (Green 2011; Mansergh and Heinze 2019). Just a few years ago, it would have been difficult to believe that such an abundant and iconic insect – with 17 000 aestivating individuals crowding onto each square metre of alpine cave wall, in untold numbers of caves and boulder fields across the great expanse of the Australian Alps – could ever be threatened. But our analysis has revealed that Bogong moths have been slowly declining for the last 40 years, and the latest drought has made it glaringly obvious that in highly unfavourable conditions the Bogong moth population can actually crash. Hopefully, their numbers will rise again as conditions improve, but our analysis, as well as that of Mansergh and Heinze (2019), suggest that further research is urgently needed. Regular quantitative monitoring of Bogong moths during their migration (e.g. using light‐trapping, vertical radar and lidar methods), during aestivation in alpine caves and during development in their breeding areas, coupled with long‐term and frequent temperature and air/soil humidity readings in these locations, is greatly warranted. Moreover, the currently unknown effects of neonicotinoid insecticides on the health of Bogong moth breeding populations should be urgently investigated.

The Bogong moth is an iconic and culturally significant Australian insect whose remarkable highly directed migration (Warrant *et al.* 2016), and its use of stellar and magnetic compass cues for nocturnal navigation (Dreyer *et al.* 2018; Adden *et al.*, in preparation), places it on‐par with its better‐known diurnal counterpart in North America, the Monarch butterfly *Danaus plexippus* (Reppert and de Roode 2018). This, in addition to its importance for the health of the alpine ecosystem (Green 2011; Mansergh and Heinze 2019), and its significance as a food source for critically endangered alpine marsupials such as the Mountain pygmy possum (Gibson *et al.* 2018; Mansergh and Heinze 2019), makes the Bogong moth both unique and exceptional among Australian insects, and worthy of every effort to ensure its long‐term survival and protection.
